# Prediction of Margin of Gait Stability by Using Six-DoF Motion of Pelvis

**DOI:** 10.3390/s24227342

**Published:** 2024-11-18

**Authors:** Tomohito Kuroda, Shogo Okamoto, Yasuhiro Akiyama

**Affiliations:** 1 Department of Computer Science, Tokyo Metropolitan University, 6-6, Asahigaoka, Hino 191-0065, Japan; black.core0415@gmail.com; 2 Faculty of Textile Science and Technology, Shinshu University, 3-15-1 Tokida, Ueda 386-8567, Japan; akiyama_yasuhiro@shinshu-u.ac.jp

**Keywords:** gait stability, postural stability, motion capture, inertial measurement unit, time-series data, principal motion analysis

## Abstract

Unstable gait increases the risk of falls, posing a significant danger, particularly for frail older adults. The margin of stability (MoS) is a quantitative index that reflects the risk of falling due to postural imbalance in both the anterior-posterior and mediolateral directions during walking. Although MoS is a reliable indicator, its computation typically requires specialized equipment, such as motion capture systems, limiting its application to laboratory settings. To address this limitation, we propose a method for estimating MoS using time-series data from the translational and angular velocities of a single body segment—the pelvis. By applying principal motion analysis to process the multivariate time-series data, we successfully estimated MoS. Our results demonstrate that the estimated MoS in the mediolateral direction achieved an RMSE of 0.88 cm and a correlation coefficient of 0.72 with measured values, while in the anterior-posterior direction, the RMSE was 0.73 cm with a correlation coefficient of 0.87. These values for the mediolateral direction are better than those obtained in previous studies using only the three translational velocity components of the pelvis, whereas the values for the anterior direction are comparable to previous approaches. Our findings suggest that MoS can be reliably estimated using six-axial kinematic data of the pelvis, offering a more accessible method for assessing gait stability.

## 1. Introduction

Unstable gait can lead to falls and associated injuries, posing a significant risk, particularly for older adults. By identifying a person’s fall risk in advance, interventions from therapists and exercise trainers may be implemented. Such interventions include, for example, indoor exercises and Tai Chi [1]. Gait stability indices quantitatively measure walking stability and are valuable in assessing fall risk. The margin of stability (MoS), a type of gait stability index based on biomechanical principles [2,3], can be applied to both normal and pathological walking. The MoS is considered a gait stability index with high construct validity [4]. Furthermore, its concurrent validity with the number of falls experienced by individuals in the past year and the score of a balance evaluation test has been reported [5]. Therefore, MoS can be used to identify and warn individuals at high risk of falling.

To compute MoS, the position and velocity of the body’s centre of mass, as well as the positions of the feet, are required, which necessitates the use of measurement devices like motion capture systems. For MoS to be used outside of a laboratory setting, it is desirable to use data that can be measured with smaller devices, such as inertial measurement units (IMUs). IMUs, which are built into smartphones and smartwatches, are well-suited for collecting data in daily life. Therefore, IMUs can be more easily used by individuals compared to motion capture systems. While IMUs have been useful for measuring gait parameters such as walking speed, cadence, and step length [6,7,8,9,10,11], or classification of gait pattern [12,13,14,15], estimating MoS from a single IMU has not yet been realized.

To achieve the objective of estimating MoS using IMUs attached to the human body, authors have proposed utilizing time-series data that can be obtained from IMUs [16,17]. This study expands these previous approaches. Iwasaki et al. [16] demonstrated that MoS in the mediolateral direction can be estimated using time-series data of translational velocities at the centre of mass (CoM). The estimated MoS showed a correlation coefficient of 0.55 with the observed MoS. Liu et al. [17] concluded that translational velocities measured from a bony landmark on the pelvis provided the most accurate estimation of MoS in the anterior and mediolateral directions among ten different body landmarks.

These studies demonstrate that MoS, which originally requires at least the position and motion data of three points on the body—namely, the CoM and both feet—can be estimated from the motion data of a single point. During normal walking, different parts of the body move in coordination [18,19,20,21]. This means that the motion information of one body part can infer the motion of another body part. For example, it is possible to estimate the position of foot placement to some extent from the velocity of the CoM [22,23]. Hence, it is worth addressing the challenge of estimating MoS from the motion data of a single body part using statistical methods.

The purpose of this study is to improve the accuracy of MoS estimation by using six-axis velocity data from a single body part, whereas previous approaches have used only three-axis translational velocities. Given that previous studies have demonstrated the usefulness of motion data from the pelvis [16,17] and considering the capabilities of an IMU, we aim to refine MoS estimation by utilizaing time-series data of the pelvis’s translational velocities and angular velocities in three directions, totaling six degrees of freedom. Based on the nature of statistical learning, providing more motion data is expected to improve the accuracy of MoS estimation. If the usefulness of six degrees of freedom motion data is confirmed, this will establish the foundation for a more accessible fall risk estimation technology using a single IMU.

It should be noted that we use an open database of gait motions of healthy Japanese individuals [24] in this study. The data were recorded using an optical motion capture system, not by IMUs. We calculated the six-axial velocities of a pelvis based on the its position and angles measured by a camera system. This approach is called as a virtual IMU [25].

## 2. Gait Data from Open Database

In this study, we used three-dimensional coordinate information of multiple body features during walking, which is contained in the AIST Gait Database [24]. Participants walked barefoot along a 10 m straight path at their own comfortable speed, with approximately one and a half walking cycles recorded in the middle of the path. An optical motion capture system (Vicon MX System and Vicon Nexus, Vicon Motion Systems Ltd., Oxford, UK) recorded the positions of markers attached to various bony landmarks at 200 Hz. The recorded positions were smoothed using a low-pass filter with a cutoff frequency of 6 Hz.

**Figure 1:**
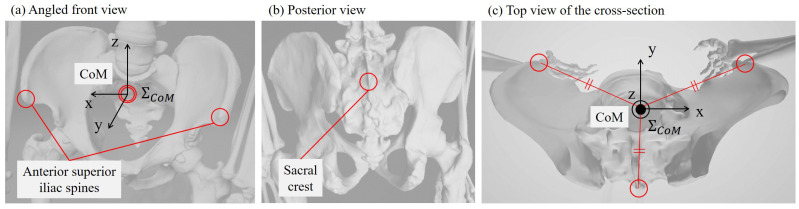
Positions of the three referenced features of the pelvis: left and right anterior superior iliac spines, and the sacral crest (centre of the left and right posterior superior iliac spines). Adapted from [28].

The centre of the second and third metatarsal points was treated as the toe, that is, the position of the base of support: $x_{\mathrm{bos}}$. We approximated the CoM [26] by the centre of three markers attached to the pelvis: the left and right anterior superior iliac spines (ASIS) and the sacral crest (the midpoint between the left and right posterior superior iliac spines), as shown in Figure 1. Following Davis et al. [27], the motion of the pelvis was calculated using the rigid body formed by these three markers.

The local coordinate system was defined as follows. The anterior (walking) direction was determined based on two successive steps. The vector connecting the positions of the CoM at two successive moments of left heel contact was defined as the anterior direction. The direction perpendicular to the anterior direction and parallel to the ground was defined as the mediolateral direction. The upward direction was aligned with the direction of the gravitational force.

Samples from 60 healthy Japanese participants (30 males and 30 females), aged 60 to 78 years, were used in this study. A total of 600 steps were analysed, with 10 steps per participant, among which 300 steps began with left heel contact.

## 3. Margin of Stability (MoS)

The MoS indicates the margin against the loss of postural balance during walking at each time point [2,3]. Although MoS can be computed for all-round directions [29], the values for the mediolateral and anterior-posterior directions have been most commonly discussed [30,31,32]. In this study, we compute the MoS for the anterior and mediolateral directions.

**Figure 2:**
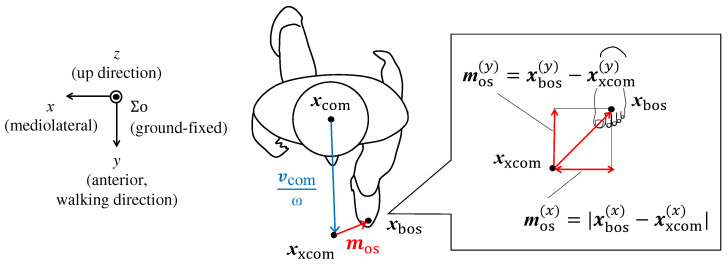
Margin of stability on the *x*–*y* plane. Distance between the extrapolated centre of mass ($x_{\mathrm{com}}$) and base of support ($x_{\mathrm{bos}}$). $x_{\mathrm{com}}$ is the centre of body mass. Adapted from [33].

To evaluate postural balance, the distance between the CoM and the edge of the base of support (BoS) is used. The MoS is the dynamic extension of this CoM-BoS distance. As shown in Figure 2, the *x* and *y* directions represent the mediolateral and anterior directions, respectively. MoS is defined as the distance between the endpoints of the BoS and the extrapolated centre of mass (XCoM). The BoS is determined by the foot positions, and during the double support phase, the foot in the direction of the CoM velocity vector is considered. The XCoM is the prospective position of the CoM [2,3]. The position vector of the XCoM on the *x*–*y* plane, which is parallel to the ground, is estimated using the velocity vector of the CoM ($v_{com}$) [2]:

(1) $$\begin{array}{ccc} x_{\mathrm{xcom}} & = & x_{\mathrm{com}}+\frac{v_{\mathrm{com}}}{\omega }, \end{array}$$

(2) $$\begin{array}{ccc} \omega & = & \sqrt{\frac{g}{l}}, \end{array}$$

where $x_{\mathrm{xcom}}$ is the position vector of the prospective CoM. For the derivation of the XCoM, the human body is approximated as an inverted pendulum, with the length of the support arm being the height of the CoM from the floor, *l*. ω represents the natural frequency of an inverted pendulum, where *g* is the gravitational acceleration. These vectors refer to a ground-fixed coordinate system ($\Sigma_{0}$).

**Figure 3:**
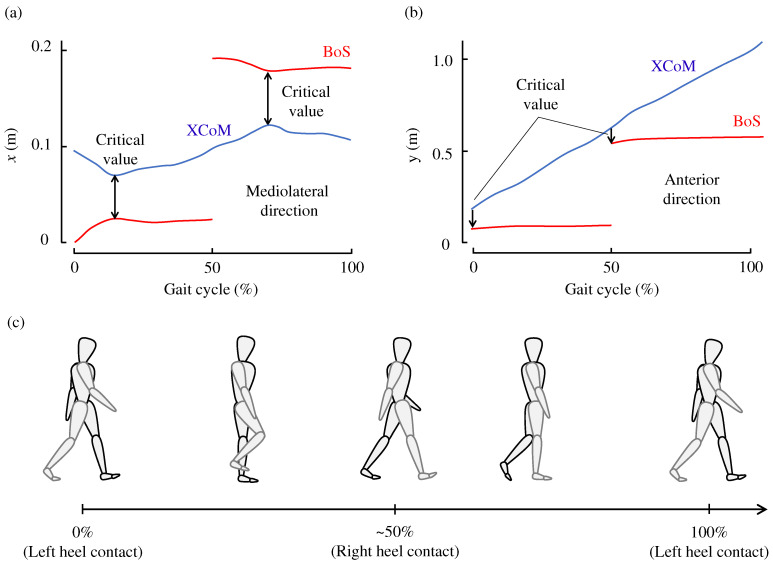
Example of the time evolution of XCoM and BoS. (**a**) In mediolateral (*x*) direction, (**b**) in anterior (*y*) direction. Adapted from [33]. (**c**) Gait cycle starting from the left heel contact.

The MoS can be computed at any time during walking. Figure 3a,b illustrates the transition of the XCoM and BoS in the mediolateral and anterior directions, respectively, throughout a gait cycle. The gait cycle is defined here as two successive steps, with one complete stride representing 100% of the cycle. At 0% of the gait cycle, the left foot touches the ground, initiating the double support phase. The right foot then leaves the ground and touches the ground again at approximately 50% of the gait cycle. Afterward, the left foot leaves the ground and recontacts the ground at 100% of the gait cycle.

The moment exhibiting the minimum XCoM-BoS distance during the gait cycle represents the most unstable timing. Therefore, when discussing mediolateral MoS, the minimum value is commonly considered [16,33,34,35,36]. In this study, we also refer to the minimum mediolateral MoS values. The larger the mediolateral MoS, the greater the postural margin in the mediolateral direction. The mediolateral MoS is defined as:

(3) $$\begin{array}{c} m_{\mathrm{os}}^{\left(x\right)}=\left|x_{\mathrm{bos}}^{\left(x\right)}-x_{\mathrm{xcom}}^{\left(x\right)}\right| \end{array}$$

where the superscript (x) indicates the *x*-component of the vector. The mediolateral MoS is the absolute distance between the XCoM and BoS in the mediolateral direction. This is primarily because the mediolateral MoS remains positive during normal walking unless the individual loses balance. In other words, the XCoM is typically positioned on the medial side of the BoS. This formulation is also convenient as the sign remains consistent even when the leading foot switches. The minimum value is typically observed at around 10% or 60% of the gait cycle [33]. At approximately these points in the cycle, the double support phase ends and the single support phase begins [37,38].

The anterior MoS is calculated using the following equation:

(4) $$\begin{array}{c} m_{\mathrm{os}}^{\left(y\right)}=x_{\mathrm{bos}}^{\left(y\right)}-x_{\mathrm{xcom}}^{\left(y\right)}. \end{array}$$

Unlike the mediolateral MoS, the anterior MoS can be either positive or negative. It is positive when the XCoM is behind the BoS, often due to slow walking speeds, and negative when the XCoM moves in front of the BoS, as depicted in Figure 3b. Similar to the mediolateral MoS, a larger anterior MoS value indicates greater postural stability in the anterior direction.

Following other studies [17,35,39], we analyse the anterior MoS values at the moment of heel contact. At this point, postural stability in the forward direction is crucial, as it marks the initial phase of body support before the next step moves forward to prevent falling. Therefore, this study discusses anterior MoS at the point of heel contact.

## 4. Principal Motion Analysis

The PMA is a set of analysis methods for multivariate time-series data [40]. These methods are broadly divided into unsupervised and supervised approaches. The unsupervised method, also known as spectrum decomposition, has been applied in fields such as the synergy analysis of human body motions [18,19,20,21]. The supervised method is an extension of partial least squares regression [40]. It decomposes a sample containing multivariate time-series data into several principal motions, each of which is also defined by multivariate time-series data. These principal motions are orthogonal to one another, and any sample can be approximated as a linear combination of these principal motions, weighted by values called scores. The principal motions are determined to maximize the covariances between these scores and the objective scalar values. The main advantage of PMA is the ease of interpreting the principal motions, as the samples are linearly decomposed into multiple principal motions, allowing for relatively straightforward interpretation of how each principal motion contributes to the determination of the objective value.

**Figure 4:**
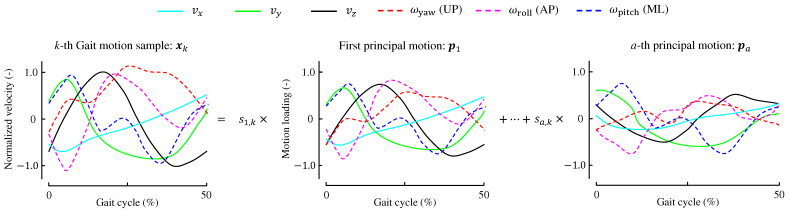
Scheme of the principal motion analysis. The motion sample $x_{k}$, composed of time-series six-axis velocities, is decomposed into several principal motions, each orthogonal to the others. The value $s_{k}$ represents the weighting factor, or score, associated with each principal motion. AP, ML, and UP indicate anterior-posterior, mediolateral, and vertical directions, respectively.

As shown in Figure 4, this study uses the time-series data of six-axis velocities, which include three-axial translational velocities and three-axial angular velocities, of the pelvis as explanatory variables. Each principal motion consists of time-series data of these 6-axis velocities. The critical mediolateral and anterior MoS values are used as objective variables. In other words, the pelvis motion is employed to predict the MoS values.

Moreover, as illustrated in Figure 3, the critical mediolateral and anterior MoS values can be defined for each step or half of a gait cycle, that is, during 0–50% or 50–100% of the gait cycle. Therefore, we predict MoS values using motion data from each step. For the motion data corresponding to the gait cycle from 50–100%, we inverted the signs of the *x*-axial velocity and roll (rotation around the anterior-posterior direction) and yaw (rotation around the vertical axis) angular velocities to cancel out the laterality.

It is worth noting that this adjustment—estimating MoS based on data from half of the gait cycle—slightly improved the overall estimation performance. However, this improvement did not affect the main conclusions of the study.

Here, the PMA for estimating MoS values from the six-axis motion data is formulated. The translational velocity along the three axes of the pelvis in the *k*-th trial ($k\in \{1,\cdots ,k^{\prime }\}$) for direction *j*(j∈{x,y,z}) is expressed as:

(5) $$\begin{array}{c} v_{j,k}={\left(v_{j,k,0},\cdots ,v_{j,k,50}\right)}^{⊤}. \end{array}$$

This vector contains the velocities at each percentage, that is, from 0% to 50% of a gait cycle. Both steps starting with the left and right heel contacts are denoted as cycles of 0–50%. Similarly, the triaxial angular velocities of the pelvis are expressed as:

(6) $$\begin{array}{c} \omega_{l,k}={\left(\omega_{l,k,0},\cdots ,\omega_{l,k,50}\right)}^{⊤} \end{array}$$

where *l* indicates the direction (l∈{roll,pitch,yaw}). These translational and angular velocities are normalised (using the *z*-score) across all trials and participants at each percentage.

The vector of explanatory variables is then expressed as:

(7) $$\begin{array}{c} x_{k}={\left({v_{x,k}}^{⊤},{v_{y,k}}^{⊤},{v_{z,k}}^{⊤},{\omega_{\mathrm{roll},k}}^{⊤},{\omega_{\mathrm{pitch},k}}^{⊤},{\omega_{\mathrm{yaw},k}}^{⊤}\right)}^{⊤}. \end{array}$$

The size of $x_{k}$ is 306 (=51×6). The number of samples is $k^{\prime }$, and all the time-series gait motions are combined into a matrix $X\in R^{k^{\prime }\times 306}$:

(8) $$\begin{array}{c} X=\left[\begin{array}{c} x_{1}^{⊤}\\ \vdots \\ x_{k^{\prime }}^{⊤} \end{array}\right]. \end{array}$$

Let $y\in R^{\left(k^{\prime }\times 1\right)}$ be the vector composed of the critical MoS values along the mediolateral (*x*) or anterior (*y*) directions:

(9) $$\begin{array}{c} y={\left(m_{\mathrm{os},k}^{\left(x\;\mathrm{or}\;y\right)}|k\in \left(1,\cdots ,k^{\prime }\right)\right)}^{⊤}. \end{array}$$

The following computation is performed separately for the MoS values in the mediolateral and anterior directions. X is decomposed into a set of principal motions, that is, a set of base vectors. Letting *a* be the number of principal motions, the explanatory and objective variables are decomposed as follows:

(10) $$\begin{array}{c} X=\sum_{n=1}^{a}s_{n}p_{n}^{⊤}+E, \end{array}$$

(11) $$\begin{array}{c} y=\sum_{n=1}^{a}q_{n}s_{n}+e, \end{array}$$

where $s_{n}={\left(s_{n,1},\cdots ,s_{n,k^{\prime }}\right)}^{⊤}$ and $p_{n}={\left(p_{n,1},\cdots ,p_{n,306}\right)}^{⊤}$ are the vectors of scores and principal motions, respectively. y is predicted by the linear summation of the scores with coefficients $q_{n}\in R$, and E and e are the matrix and vector of prediction errors, respectively.

Each principal motion score is determined such that its covariance with the objective variable, that is, y, is maximized. The principal motion score of the *n*-th principal motion is determined as:

(12) $$\begin{array}{c} s_{n}=X_{n}\left(\frac{X_{n}^{⊤}y_{n}}{∥X_{n}^{⊤}y_{n}∥}\right), \end{array}$$

where ∥·∥ is the $L^{2}$ norm. Using this $s_{n}$, $p_{n}$ and $q_{n}$ are computed as:

(13) $$\begin{array}{c} p_{n}=\frac{X_{n}^{⊤}s_{n}}{s_{n}^{⊤}s_{n}}, \end{array}$$

(14) $$\begin{array}{c} q_{n}=\frac{y^{⊤}s_{n}}{s_{n}^{⊤}s_{n}}. \end{array}$$

The *n*-th (n∈{1,⋯,a}) principal motion accounts for the part that remains unexplained by the previous principal motions. To compute the *n*-th principal motion, the portions explained by up to the (n−1)-th principal motions are subtracted from X and y:

(15) $$\begin{array}{c} X_{n}=X-\sum_{u=1}^{n-1}s_{u}p_{u}^{⊤}, \end{array}$$

(16) $$\begin{array}{c} y_{n}=y-\sum_{u=1}^{n-1}q_{u}s_{u}. \end{array}$$

Note that $X_{1}=X$ and $y_{1}=y$. The principal motions are computed *a* times using these formulae. When the number of principal motions is *a*, y is estimated using the following formula:

(17) $$\begin{array}{c} \overset{ʌ}{y}=\sum_{n=1}^{a}q_{n}s_{n}. \end{array}$$

Note that these processes, from (10) to (17), are performed separately for the mediolateral and anterior directions of MoS.

The number of principal motions (*a*) is a hyperparameter determined by cross-validation. For this purpose, all motion samples are randomly divided into five groups for five-fold cross-validation. One of the groups forms the test dataset, while the remaining groups form the training dataset. The estimation model is established using the training data, and the loadings $p_{n}$ are computed. The scores for the test data are obtained as:

(18) $$\begin{array}{c} s_{n}^{\left(\mathrm{test}\right)}=X^{\left(\mathrm{test}\right)}p_{n}, \end{array}$$

where $X^{\left(\mathrm{test}\right)}$ contains the velocity values of the test dataset. Using these scores $s_{n}$ and Equation (17), the critical MoS values for the test data are estimated. We then calculate the root mean squared errors (RMSE) between the estimated MoS and the actual MoS values. After repeating the estimation such that all groups are tested once, we select the number of principal motions *a* that results in the smallest RMSE.

## 5. Gait Parameters

We calculated several gait parameters to aid in the interpretation of the principal motions. These include the velocity of the CoM, step width, step length, cadence, swing duration, and minimum foot clearance.

The velocities of the CoM are the maximal absolute velocities along the mediolateral and anterior directions. Step width is defined as the distance between the left and right feet in the mediolateral direction at the moment when both heels are on the ground. Step length refers to the anterior distance traveled within a step. Cadence is the number of steps per minute. The single support phase is the portion of the gait cycle during which only one foot is in contact with the ground, supporting the body. Minimum foot clearance is the distance between the sole of the foot and the ground when the swinging foot is parallel to the ground. These gait parameters have been studied in relation to MoS in previous research [16,32,33,34,35,36,39,41,42,43].

The means and standard deviations of MoS, gait parameters, as well as height and weight, are presented in Table 1. We calculated the correlation coefficients between these gait parameters and the principal motion scores. For this analysis, the velocities of the CoM, step width, and stride length were normalised by dividing by each participant’s height.

## 6. Results

Table 2 shows the correlation coefficients and RMSEs between the observed and estimated critical mediolateral MoS values with different numbers of principal motions used for the estimation. It presents the means and standard deviations of the correlation coefficients across five iterations of the five-fold cross-validation. Although the primary focus of this study is on the accuracy of MoS estimation using six-axis motion data, for comparison, the correlation coefficients and RMSE values are also provided for the three-axis conditions, where either the three-axis translational velocities or angular velocities were used for MoS estimation.

**Figure 5:**
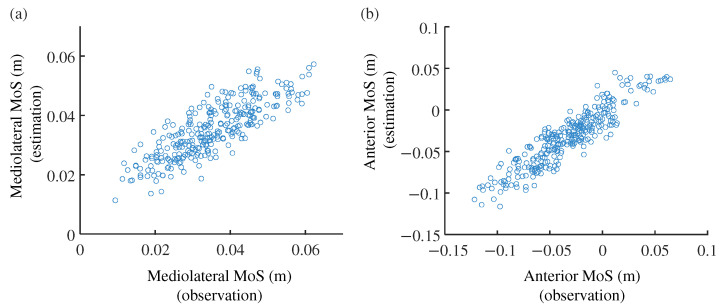
Scatter plot of estimated and observed critical MoS values. (**a**) Mediolateral MoSs. Correlation coefficient between the estimated and observed MoS is r=0.718. (**b**) Anterior MoSs. r=0.865.

For the mediolateral direction, the correlation coefficient was highest when the number of principal motions was three: r=0.718±0.04, which is just above the threshold between high and moderate correlation [44]. Regarding the RMSE values, conditions with three or more principal motions were comparable, with RMSE = 0.88 cm. Therefore, we adopted three principal motions (a=3) for mediolateral MoS estimation, as the model with a=3 is simpler than those with higher *a* values. Figure 5a shows the scatter plot of the observed and estimated mediolateral MoS values for this condition.

Including a fourth or fifth principal motion did not improve estimation accuracy when six-axis data were used. These additional principal motions even reduced accuracy when only three-axis data were used. This suggests that the fourth and later principal motions may capture motion information specific to the training data, rather than common patterns across all samples, thus reducing generalisation capability.

When the PMA model was established using all the samples, the linear model to estimate MoS from the principal motion scores was as follows:

(19) $$\begin{array}{c} {\hat{m}}_{\mathrm{os}}^{\left(x\right)}=0.11s_{1}+0.050s_{2}+0.030s_{3}. \end{array}$$

The weighting coefficients for the first principal motion scores were the largest, followed by those for the second and third principal motion scores.

**Figure 6:**
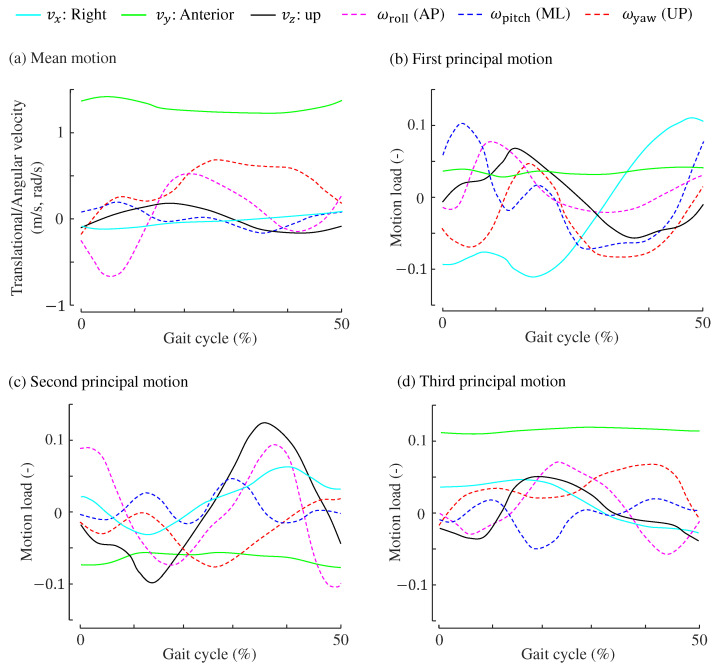
Principal motions of the mediolateral MoSs. (**a**) Mean translational and angular velocities across all the samples. (**b**) First principal motion. (**c**) Second principal motion. (**d**) Third principal motions.

Correlation coefficients between the principal motion scores and gait parameters are provided in Table 3, where only significant correlation coefficients with p<0.05 are listed. Further, Figure 6 shows the three principal motions as well as the average motion of the six-axial pelvic velocities. The effects of these principal motions on the mediolateral MoS are discussed in Section 7.

Table 4 shows the correlation coefficients for the critical anterior MoS values. Using the six-axis motion data, the average correlation coefficients were equally high when the number of principal motions was three to five. Correspondingly, the RMSE values were nearly identical for these three conditions. Among them, the simplest model was chosen: a=3. It is noteworthy that these indices are comparable to those obtained using the three translational velocities at a=3, suggesting that the translational velocities alone are sufficient and that angular velocities do not enhance the estimation accuracy for the anterior direction. Figure 5b shows the scatter plot of the observed and estimated anterior MoS values with a=3.

Using all the motion samples, the linear regression model for estimating the critical anterior MoS values was determined as follows:

(20) $$\begin{array}{c} {\hat{m}}_{\mathrm{os}}^{\left(y\right)}=0.073s_{1}+0.069s_{2}+0.037s_{3}. \end{array}$$

As with the mediolateral MoS, the weighting values of the scores were largest for the first principal motion, followed by the second and third principal motions.

**Figure 7:**
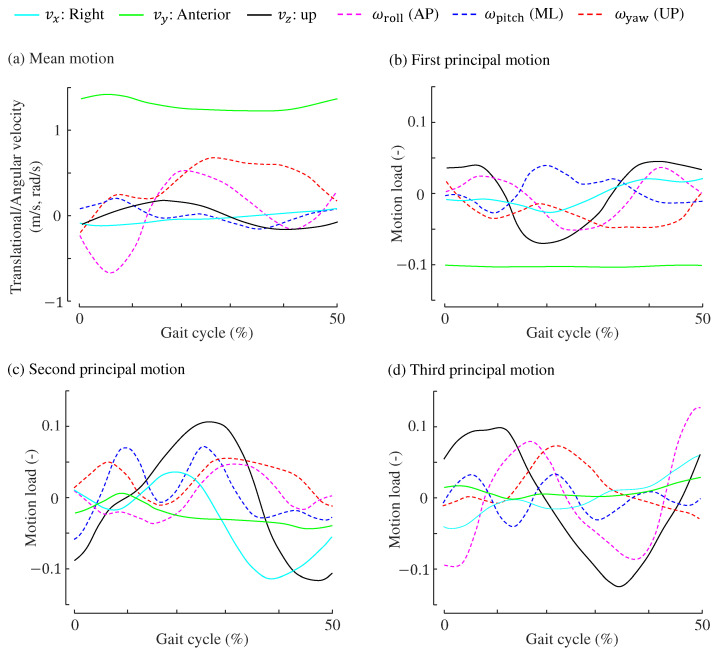
Principal motions used for estimating the anterior MoS. (**a**) Mean translational and angular velocities (same as Figure 6a). (**b**) First principal motion. (**c**) Second principal motion. (**d**) Third principal motion.

Figure 7 shows the three principal motions used for estimating the anterior MoS. Table 5 shows the correlation coefficients between the gait parameters and principal motion scores.

## 7. Discussion

### 7.1. Interpretation of Principal Motions for the Mediolateral MoS

As mentioned in Section 1, a key advantage of principal motion analysis is the explainability of the established model. We attempt to interpret the principal motions for both the mediolateral and anterior MoS values. A reasonable interpretation of these principal motions further validates the analysis performed using PMA.

Here, we interpret the meanings of the three principal motions used to estimate the mediolateral MoS values. To aid in the interpretation of the first to third principal motions, we refer to Table 3, which shows the correlation coefficients between the principal motion scores and gait parameters. The fourth and fifth principal motions, which were not used in this study and did not contribute to improving the estimation accuracy of MoS, are difficult to interpret with clear rationale, partly because their scores did not show substantial correlations with gait parameters.

#### 7.1.1. First Principal Motion for Mediolateral MoS

The scores of the first principal motion were positively correlated with step width (r=0.65). Therefore, gait samples with high scores along the first principal motion are likely to exhibit a wider step width. As step width increases, the mediolateral MoS also increases because the boundary of the base of support moves further from the CoM, consistent with the definition of MoS. Previous studies have similarly concluded that a wider step width enhances mediolateral stability [33,36,45]. Additionally, the scores for the first principal motion show a positive correlation (r=0.28) with CoM velocity in the mediolateral direction, which aligns with findings that changes in mediolateral velocity are associated with changes in step width [46].

The first principal motion exhibits a strong correlation (r=0.74) with mediolateral MoS, but a slight negative correlation (r=−0.19) with anterior MoS, as shown in Table 3. Other principal motions are also correlated with both types of MoS, suggesting that mediolateral and anterior MoS values are not independently determined, which is a characteristic often observed in older adults [33].

It is important to note that PMA decomposes any gait sample into three principal motions along with the average of all samples. Therefore, each principal motion illustrates how a specific sample deviates from the mean. As shown in Figure 6b, the loadings for the mediolateral and upward velocities are in phase with the mean values in Figure 6a, indicating that gait samples with higher scores on the first principal motion show more pronounced variations in these velocities. In contrast, the angular velocity along the yaw direction (rotation around the vertical axis) in the first principal motion is almost in the opposite phase to that of the average motion. This suggests that this principal motion suppresses the yaw motion of the pelvis. Given that pelvis yaw motion is influenced by the position of the leading foot [47], this reduction may result from the wider step width associated with the first principal motion.

#### 7.1.2. Second Principal Motion for Mediolateral MoS

The second principal motion score showed positive correlations with walking parameters related to the mediolateral direction (mediolateral velocity and step width). Thus, as this score increases, the mediolateral MoS tends to be larger, with a correlation coefficient of r=0.30. Furthermore, higher scores are associated with smaller step lengths and slower walking speeds. As shown in Figure 6c, the loadings for the anterior velocity $v_{y}$ are negative throughout the 0–50% gait cycle. Additionally, the loading for yaw (rotation around the vertical axis) is nearly in antiphase with the average yaw motion, indicating that gaits with higher second principal motion scores exhibit reduced pelvis yaw motion and shorter step lengths.

Geometrically, as step length decreases, the vertical movement of the CoM diminishes, resulting in a smaller absolute value of the centre of mass’s vertical velocity. The loadings for vertical velocity in the second principal motion are in antiphase with the mean vertical velocity shown in Figure 6a, suggesting that gait motions characterized by the second principal motion involve less vertical movement.

Distinct gait patterns associated with the second principal motion indicate a slower walking speed in the anterior direction (r=−0.29) and shorter step length (r=−0.48). This observation aligns with findings from earlier studies [32,33,39,43], suggesting that older individuals maintain mediolateral stability during slow-paced walking. It should be noted, however, that slow walking does not necessarily increase postural stability [35,48].

#### 7.1.3. Third Principal Motion for Mediolateral MoS

Unlike the first and second principal motions, the third principal motion showed no correlation with step width and had a weak negative correlation of r=−0.15 with mediolateral CoM velocity. In situations where step width remains constant and mediolateral velocity decreases, the XCoM shifts medially, leading to an increase in mediolateral MoS. Therefore, it is understandable how this principal motion contributes to the mediolateral MoS.

Interestingly, the third principal motion is evidently linked with gait parameters in the anterior direction: step length (r=0.43), anterior velocity (r=0.67), and cadence (r=−0.68). Hence, this principal motion exhibits a strong negative correlation with the anterior MoS (r=−0.80), indicating postural instability in the anterior direction, whereas its effect on the mediolateral MoS is relatevely minor (r=0.22).

### 7.2. Interpretation of Principal Motions to Estimate Anterior MoS

#### 7.2.1. First Principal Motion for Anterior MoS

The first principal motion score correlates with gait speed, step length, and cadence, with correlation coefficients of −0.85, −0.64, and 0.70, respectively. This principal motion is characterized by slow gaits with shorter step lengths, where higher cadences are typically used to compensate for reduced walking speed.

As walking slows, the XCoM moves closer to the CoM, resulting in an increase in MoS. Meanwhile, since step length is also reduced when the first principal motion score is high, the distance between the BoS and CoM becomes smaller, suggesting lower postural stability. However, the effect of the slower walking speed may outweigh that of the shorter step length, leading to increased stability in the anterior direction. As a result, the first principal motion strongly relates to the anterior MoS, with no clear impact on the mediolateral MoS.

As illustrated in Figure 7b, the loadings for vertical velocity, yaw, roll, and pitch are largely in antiphase with the average motions. Thus, gaits associated with the first principal motion involve reduced pelvic motion.

#### 7.2.2. Second Principal Motion for Anterior MoS

Interpreting the second principal motion is challenging because, unlike other motions, it does not display any strong correlations with gait parameters, as shown in Table 5. The second principal motion score shows a moderately positive correlation with anterior MoS (correlation coefficient of 0.35), but a negative correlation with mediolateral MoS (r=−0.37), suggesting that gaits characterized by the second principal motion may sacrifice mediolateral stability for anterior stability.

One notable feature of the second principal motion is its negative correlation with swing duration (r=−0.31). Additionally, as shown in Figure 7c, the loadings for CoM upward velocity lag behind the average motion. Although these characteristics distinguish the second principal motion from others, we were unable to determine how these features contribute to improving anterior stability while compromising mediolateral stability.

#### 7.2.3. Third Principal Motion for Anterior MoS

The prominent features of the third principal motion are the moderately positive correlation with the step length (0.31) and cadence (0.34). In a long stride, the BoS moves further forward, thus logically increasing the anterior MoS.

Further, this principal motion leads the phase of gait motions because the phases of the profiles shown in Figure 7d lead those of the average motions in (a) by approximately 10%.

### 7.3. General Discussion

As shown in Table 2 and Table 4, using six-axis pelvis motion allows for a more accurate or equally accurate estimation of mediolateral and anterior critical MoS values compared to three-axis motion. This trend is more pronounced for mediolateral MoS. A key question arises: why are the angular velocities of the pelvis effective for MoS estimation when only position and translational velocity vectors of body parts are used to calculate MoS? This can be attributed to the synergy or coordination between pelvis motion and lower limb movements [21,49,50,51].

For instance, Crosbie and Vachalathiti [49] found that the phasic relationships between hip flexion and pelvis angular motion are more constrained at faster walking speeds. Similarly, Langley et al. [51] reported phasic synchrony between pelvis yaw motion and hip flexion/extension in both healthy individuals and patients post-total hip arthroplasty. Thus, the angular velocities of the pelvis indirectly provide the PMA with information about lower limb movements, which are directly related to postural stability.

The efficacy of angular velocities appears to depend on the direction of the MoS. According to Table 2, angular velocities are more or equally effective as translational velocities for estimating mediolateral MoS. However, as shown in Table 4, angular velocities are less reliable than translational velocities for estimating anterior MoS. The position of foot placement can be somewhat predicted from CoM translational velocities [22,23], so in some cases, CoM translational velocities alone may suffice for MoS estimation.

Several limitations and future directions should be noted. First, while we compared six-axis and three-axis conditions for MoS estimation using PMA, we did not explore other estimation algorithms. It is likely that the superior performance of six-axis data will persist across different algorithms, but testing additional machine learning algorithms is essential to further improve estimation accuracy.

Second, the applicability of this approach to different age groups is uncertain. Gait strategies differ across age groups [33,52,53,54], suggesting that the PMA model may need to be adapted for each group.

Third, this study relied on velocity data obtained from a camera-based motion capture system, leaving the impact of IMU noise on estimation accuracy unclear. Addressing this issue will be crucial for developing more accessible methods for estimating MoS. It is also important to consider that the motion of an IMU attached to the skin over a bony landmark of the pelvis, such as the sacral crest, may not perfectly correspond to the motion of the pelvis itself. Additionally, when using a smartphone as an easily accessible IMU device, methods for secure attachment to the pelvis need to be considered. Validating the approach with a lightweight IMU equipped with wireless communication capabilities would be a practical next step for further research.

Fourth, this study’s method for estimating MoS values is limited in its applicability to symmetrical gait patterns. For individuals with pathological gait involving mediolateral asymmetry, this approach, based on assumed lateral symmetry, may result in inaccuracies. For accurate MoS estimation in asymmetrical gait, it would be necessary to analyze data from the entire gait cycle or to separately consider right and left steps.

## 8. Conclusions

This study aimed to improve the accuracy of estimating MoS during gait using six degrees of freedom motion data from a single body segment, the pelvis. By utilizing time-series data of both translational and angular velocities, we applied PMA to estimate MoS in both the mediolateral and anterior-posterior directions. The results showed that using six-axis data improved estimation accuracy for the mediolateral direction compared to previous approaches that used only translational or angular velocities. The estimated mediolateral MoS achieved an RMSE of 0.88 cm and a correlation coefficient of 0.72, outperforming earlier studies. The anterior-posterior MoS yielded an RMSE of 0.73 cm and a correlation coefficient of 0.87, comparable to those reported in earlier studies.

Our findings suggest that incorporating both translational and angular velocities provides a more comprehensive representation of body dynamics, which enhances the prediction of MoS. This method allows for a more accessible and practical approach to estimating gait stability using a single IMU, which could be integrated into wearable technologies such as smartphones and smartwatches. The ability to estimate MoS accurately outside laboratory environments opens new possibilities for fall risk assessment, particularly in elderly and frail populations.

In conclusion, this study provides a foundation for developing accessible and reliable fall risk assessment tools using wearable devices. Future research should focus on expanding the method’s applicability by employing an actual IMU and refining its accuracy across various populations and walking conditions.

## Figures and Tables

**Table 1:** Means and standard deviations of height, weight, gait parameters and MoSs in 60 elderly individuals.

Parameters	Mean ±SD
Height (cm)	159.63±7.61
Weight (kg)	60.65±9.35
Minimum foot clearance (m)	0.021±0.010
Maximal mediolateral velocity of CoM (m/s)	0.12±0.03
Maximal anterior velocity of CoM (m/s)	1.44±0.16
Step width (m)	0.15±0.03
Step length (m)	0.63±0.045
Cadence (steps/min)	117.71±9.15
Total single support phase over two steps (%)	85.63±8.86
Mediolateral MoS (m)	0.035±0.010
Anterior MoS (m)	−0.033±0.040

**Table 2:** Correlation coefficients and RMSE (cm) between the estimated and observed mediolateral MoS values for different numbers of principal motions used in the estimation. Upper row: Correlation coefficients (mean ± standard deviation) calculated over five iterations of five-fold cross-validation. Lower row: RMSE across all samples in the test dataset.

The Number of Principal Motions	6-Axis Motions	3-Axis Translational Velocity	3-Axis Angular Velocity
1	0.62±0.03	0.39±0.07	0.51±0.06
	0.94 cm	1.05 cm	0.97 cm
2	0.66±0.03	0.46±0.04	0.57±0.03
	0.93 cm	0.98 cm	0.96 cm
3	0.72±0.04	0.50±0.07	0.64±0.04
	0.88 cm	1.23 cm	0.94 cm
4	0.71±0.02	0.45±0.09	0.58±0.05
	0.88 cm	1.20 cm	0.97 cm
5	0.71±0.01	0.38±0.09	0.62±0.04
	0.88 cm	1.24 cm	0.94 cm

**Table 3:** Correlation coefficient between the principal motion scores in estimating the mediolateral MoSs and gait parameters. Significant correlation coefficients are listed (p<0.05).

	1st Principal Motion	2nd Principal Motion	3rd Principal Motion
Minimum foot clearance	-	-	0.14
Maximal mediolateral velocity of CoM	0.28	0.27	−0.15
Maximal anterior velocity of CoM	-	−0.29	0.67
Step width	0.65	0.45	-
Step length	-	−0.48	0.43
Cadence	-	-	−0.68
Single support phase	0.24	−0.20	0.32
Mediolateral MoS	0.74	0.30	0.22
Anterior MoS	−0.19	0.17	−0.80

**Table 4:** Correlation coefficients (upper row) and RMSE values (lower row) for the estimation of anterior MoS. The means and standard deviations of the correlation coefficients were computed across iterative runs of the five-fold cross-validation.

The Number of Principal Motions	6-Axis Motions	3-Axis Translational Velocity	3-Axis Angular Velocity
1	0.79±0.03	0.80±0.02	0.43±0.08
	0.83 cm	0.83 cm	1.28 cm
2	0.81±0.03	0.85±0.01	0.56±0.04
	0.82 cm	0.75 cm	0.99 cm
3	0.87±0.02	0.86±0.02	0.47±0.09
	0.73 cm	0.73 cm	1.20 cm
4	0.87±0.01	0.78±0.04	0.56±0.07
	0.73 cm	0.85 cm	0.99 cm
5	0.87±0.01	0.84±0.03	0.53±0.07
	0.74 cm	0.75 cm	1.01 cm

**Table 5:** Correlation coefficients between principal motion scores in estimating the anterior MoSs and gait parameters (p<0.05).

	1st Principal Motion	2nd Principal Motion	3rd Principal Motion
Minimum foot clearance	−0.17	0.16	-
Maximal mediolateral velocity of CoM	0.16	-	-
Maximal anterior velocity of CoM	−0.85	-	-
Step width	-	−0.26	-
Step length	−0.64	0.16	0.31
Cadence	0.70	0.17	0.34
Single support phase	−0.26	−0.31	-
Mediolateral MoS	-	−0.37	-
Anterior MoS	0.82	0.35	0.19

## Data Availability

This study used data from an open database [24].

## Abbreviations

The following abbreviations are used in this manuscript:

MoS	Margin of stability
CoM	Centre of mass
XCoM	Extended centre of mass
BoS	Base of support
